# Deep-Learning-Based Hair Damage Diagnosis Method Applying Scanning Electron Microscopy Images

**DOI:** 10.3390/diagnostics11101831

**Published:** 2021-10-03

**Authors:** Lintong Zhang, Qiaoyue Man, Young Im Cho

**Affiliations:** AI/SC Lab, Computer Engineering of Gachon University, Seongnam-si 461-701, Gyeonggi-do, Korea; zhanglintong1@naver.com (L.Z.); manqiaoyue@gmail.com (Q.M.)

**Keywords:** hair damage, image classification, deep learning, damaged cuticle layers, SEM image

## Abstract

In recent years, with the gradual development of medicine and deep learning, many technologies have been developed. In the field of beauty services or medicine, it is particularly important to judge the degree of hair damage. Because people in modern society pay more attention to their own dressing and makeup, changes in the shape of their hair have become more frequent, e.g., owing to a perm or dyeing. Thus, the hair is severely damaged through this process. Because hair is relatively thin, a quick determination of the degree of damage has also become a major problem. Currently, there are three specific methods for this purpose. In the first method, professionals engaged in the beauty service industry make a direct judgement with the naked eye. The second way is to observe the damaged cuticle layers of the hair using a microscope, and then make a judgment. The third approach is to conduct experimental tests using physical and chemical methods. However, all of these methods entail certain limitations, inconveniences, and a high complexity and time consumption. Therefore, our proposed method is to use scanning electron microscope to collect various hair sample images, combined with deep learning to identify and judge the degree of hair damage. This method will be used for hair quality diagnosis. Experiment on the data set we made, compared with the experimental results of other lightweight networks, our method showed the highest accuracy rate of 94.8%.

## 1. Introduction

**Figure 1:**
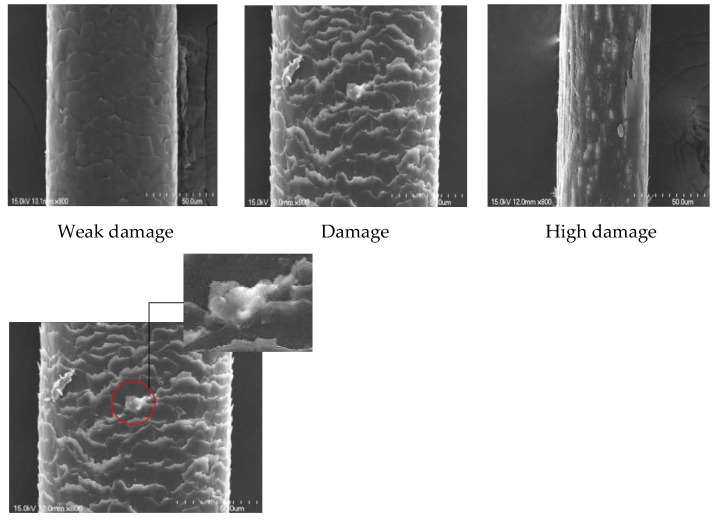
Image of degree of hair damage under SEM microscope ×800; from left to right: damage, high damage, and weak damage. The bottom image shows damaged cuticle layers.

Hair quality inspection and damage determination in beauty salons rely only on the judgment of professionals such as beauticians, which is mostly based on the tactile experience of beauticians and observations with the naked eye. However, in the absence of excessive professional experience, errors can occur when judging the quality of the hair. The current methods of judging hair damage are to determine the hair damage by detecting the moisture content, cystine content, coagulation, and/or relaxation; apply a dye absorption method, the alkali solubility, copper absorption method, or lithium bromide method [1]; or consider the absorbance and tensile strength. Ref. [2] Using these methods, we need to test the composition of many aspects in a chemical or physical experiment. Compared with our proposed method, this type of chemical and physical method for detecting hair is complicated and time-consuming. Therefore, we need a faster and simpler approach to determine the degree of hair damage. This method will be applied to portable devices in the future, and everyone can easily judge the degree of damage to one’s hair anytime and anywhere, and then decide whether to receive a perm, dye, or other beauty services. To better observe cuticle layers in the hair, we used a scanning electron microscope (SEM) and observed it at ×800. Thus, according to the form of cuticle layers (Figure 1), damaged cuticle layers (the white part) accounts for the proportion of hair. We define a lifting up of the cuticle edge and an irregular overlay of cuticles without cracks or holes as weak damage; cracks or holes due to severe lifting up of cuticle layers as damage; exposure of cortical cells and complete disappearance of cuticles as high damage. Regarding the analysis of the SEM image, the segmentation method was developed using the watershed segmentation algorithm, the global–local threshold method, Laplacian of Gaussian filter, and non-maximum suppression in [3].

We, therefore, judged that the degree of hair damage directly seen in the hair images was too simple to understand. Thus, we need to recognize and judge the degree of hair damage in the hair images obtained through observations under a microscope. In this study, we combine deep learning image classification technology for recognition and classification. 

The main contributions of this study through the use of a lightweight Convolutional Neural Network (CNN) [4] are as follows:

- Decreases the number of free parameters.
- Achieves a high classification accuracy using small datasets.
- Reduces the training time required for convergence.
- Decreases the complexity of the network, thus, enabling mobile applications.

Establishes a future research direction that will extend the applicability of the method to digital microscopy devices (for example:SVBONY SM401 Microscope Wireless Digital Microscope 50×–1000×).

Weak damage

Damage

High damage

Damaged cuticle layers

## 2. Materials and Methods

### 2.1. Datasets of SEM Images (DHI Dataset) 

**Figure 2:**
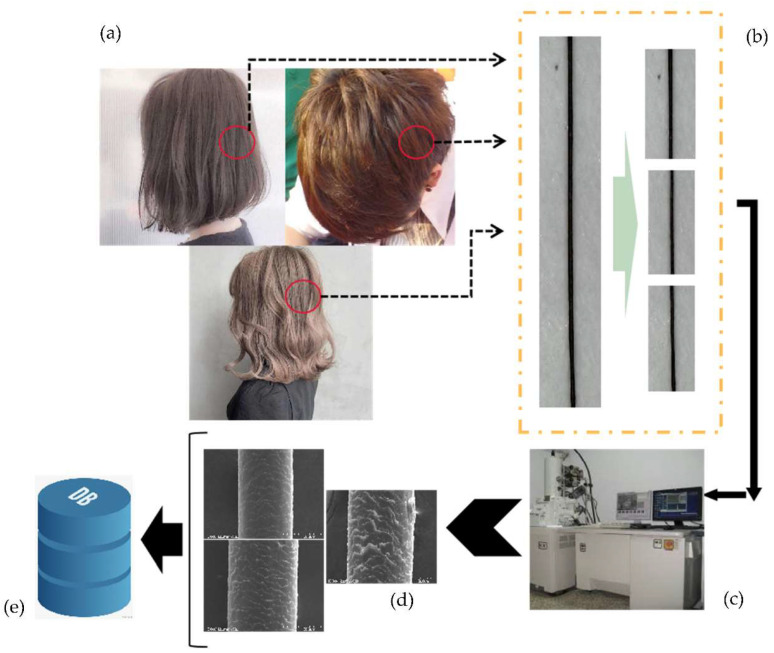
The processing of hair damage data collection, (**a**) is the many style of hair, (**b**) is the samples divided into three parts, (**c**) is the data collected on scanning electron microscope, (**d**) is the SEM image of hair, (**e**) is the many SEM hair images made into a DHI dataset.

For deep learning, it is important to choose a dataset. To identify and judge the degree of hair damage, we need to identify and classify cuticle layers in the hair images. However, according to our investigation, there are very few datasets on microscopic images of hair that clearly show the cuticle layers. For this reason, we collected and produced a dataset directly, called the damage hair image (DHI) dataset, as shown (Figure 2). The DHI dataset was collected through our own observation using SEM. During the process of data collection, we collected various hair samples for observation and image collection. The dataset contains hair samples from young to old people, from 19 to 55 years old, as well as perm and non-perm processed hair samples. Using distance from the scalp less than 1 cm, from 3 to 5 cm, and over 5 cm, we sectioned the upper, middle, and lower parts of the hair to be hair image data sample (from the root to the tip), and then contrasted and observed them under SEM. After the observation, the cuticle layers in the sample were arranged in an orderly manner. A small amount of the middle part of the cuticle layers is generally missing, whereas the cuticle layers in the lower part is largely missing, and in some areas is completely gone. However, the perm hair samples were irregularly distributed, and cuticle layers were shown to be shedding from the center. After a long period of time, we collected images through SEM, and then analyzed and organized them. The final sample data images of minor, moderate, and severe injuries numbered 286 in total. However, this number of datasets is still too low for deep learning. Therefore, we applied data expansion technology [5] including rotation, blur, and a noise increase to expand the dataset to 2900 images. The original image resolution was 640 × 480, and to make the data training of the neural network faster and more convenient, the resolution was adjusted to 224 × 224.

### 2.2. Methodology

#### 2.2.1. Convolutional Neural Networks 

**Figure 3:**
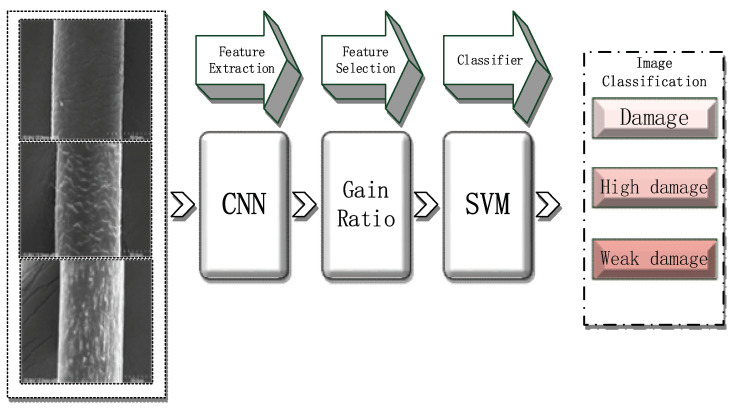
Flowchart of hair damage diagnosis.

A convolutional neural network (CNN) is a type of feedforward neural network that includes convolution calculations and has a deep structure and is a representative deep learning algorithm [6]. CNNs have representation learning capabilities and can classify input information according to their structure, and in recent years, recognition and classification networks in deep learning have become increasingly mature, including MobileNet, Googlenet, and VggNet. These networks have demonstrated a high accuracy in the recognition and classification of animals, plants, faces, and other fields. However, we need a small network for experimentation, which can be applied to mobile phones or portable devices in the future, and because our dataset is small, it is prone to a poor training and easy overfitting on large networks. We, therefore, need a smaller model than a VGG or ResNet. For greater accuracy in the classification and recognition results of hair damage, we propose a network suitable for hair classification, i.e., Hair-Diagnosis-Mobilenet (HDM-NET). In addition, we use HDM-NET to extract and select the features, and finally use an SVM [7] to classify hair damage images (Figure 3). In this study, we used an SVM for a single experiment and HDM-NET combined with multilayer perceptron (MLP) [8], random forest (RF) [9], k-nearest neighbor (KNN) [10], and the original MobileNet model combined with the classification methods to conduct multi-group control experiments. Finally, we discuss the advantages and disadvantages of the proposed method.

#### 2.2.2. Select Features

When the feature vector is generated from the image information, we choose the attributes. The significance of this choice is to eliminate unnecessary features when analyzing the image, and then reduce the computational complexity, thereby optimizing the prediction model, obtaining better results.According to [11], attribute selection technology is mainly used to identify basic information. Herein, we use the gain ratio [12] algorithm for selection. When calculating the information gain, entropy is used to measure the complexity and decision trees [13] to observe the vector attributes. In this way, the information gain is improved. It, therefore, provides a higher accuracy when spanning the tree. The dataset (D) consists of data samples with different categories (T). Equation (1) presents the classification calculation information for a given sample.

(1) $$Inca\left(D\right)=-\sum_{i=1}^{t}p_{i}\log_{2}(p_{i})$$

where 𝑝_𝑖_ is the probability of that the sample belongs to class 𝐶_𝑖_. The entropy calculation of a given attribute 𝐴*t* having 𝑣 values is shown in Equation (2),

(2) $$Entropy\left(At\right)=-\overset{t}{\underset{i=1}{\sum }}Inca\left(D\right)\frac{S1i+S2i+\ldots +Sti}{s}\;$$

where $D_{ij}$ represents the number of samples belonging to class $C_{i}$ of subset $D_{i}$. The attribute gain 𝐴*t* is represented through Equation (3):

(3)        Gain(At)=Inca(D)−Entropy(At)

Equation (4) presents the information value generated by dividing the dataset of *D* into 𝑣 partitions:

(4) $$\;\;SInca\left(D\right)=-\overset{v}{\underset{i=1}{\sum }}\left(\frac{\left|S_{i}\right|}{\left|S\right|}\right)\log_{2}\left(\frac{\left|S_{i}\right|}{\left|S\right|}\right)$$

Finally, the gain ratio is defined as the result of dividing the solution into Equation (3) by solving Equation (4). The attributes are ranked according to the values of their gain ratios, and 𝑛 attributes with highest values are selected.

#### 2.2.3. MobileNet 

**Figure 4:**
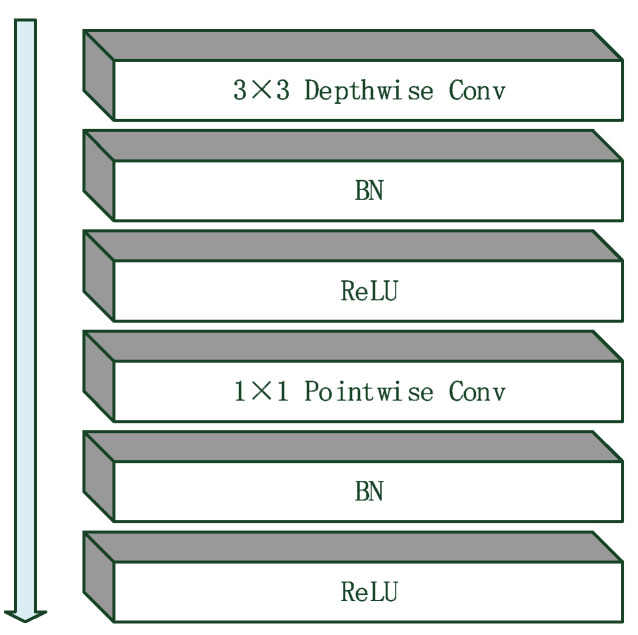
Basic structure convolution layers of MobileNet.

We selected and improved MobileNet [14], which can be used in mobile devices and small microscopes or mobile cameras to obtain and diagnose hair images. MobileNet is based on depthwise separable convolutions, which consist of two core layers: depthwise [15] and pointwise [16] convolutions. A depthwise convolution is the step of filtering the input without creating new features. Thus, the process of generating new features, called a pointwise convolution, was also applied. Finally, the combination of the two layers is called a depthwise separable convolution. This model uses depthwise convolutions to apply a single filter for each channel of input, and then uses a 1 × 1 convolution (pointwise) to create a linear combination of output from the depthwise layer. Batch normalization (BN) [17,18] and a rectified linear unit (ReLU) [19,20] were used after each convolution. (Figure 4) shows the architecture of MobileNet, which consists of a convolution layer, a depthwise convolution layer followed by a BN layer and an ReLU layer, a pointwise convolution layer, a BN and ReLU layer, a global average pooling layer, a reshape layer, a dropout layer, a convolutional layer, a softmax layer, and a reshape layer. This model contains approximately four million parameters, which is a much smaller number than those of the other models. The structure of MobileNet (depthwise + pointwise convolutions).

#### 2.2.4. Hair-Diagnosis Mobilenet (HDM-NET) 

**Figure 5:**
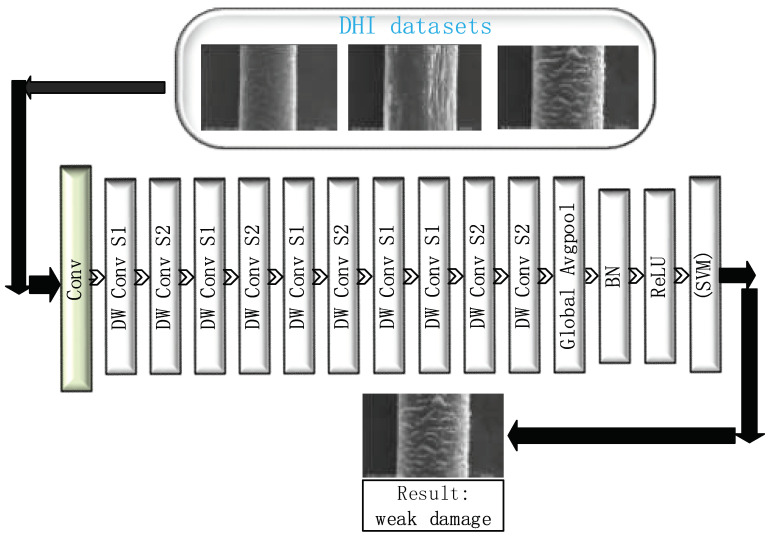
Proposed model architecture: Hair-Diagnosis Mobilenet (HDM-NET + SVM) and process of training the CNN model (HDM-NET), convolution (Conv), depthwise (DW), and batch normalization (BN). The classifier can also be changed for RF, MLP, and KNN.

Because our dataset is relatively small, when testing the original MobileNet model, the classification results were unsatisfactory. Therefore, we made some improvements based on the MobileNet model. We removed three layers from the five layers of depthwise convolution in MobileNet. The purpose here was to reduce the redundant parameters of the model with almost no effect on the results. We then changed the final average pooling to global pooling [21] and added batch normalization. Relu will cause some neurons to output a 0, resulting in a sparsity of the network, reducing the interdependence between parameters, and alleviating the occurrence of an overfitting. We, therefore, used Relu. Finally, the output [22,23] applies an SVM (Figure 5). After such changes, this model achieved good results in our DHI dataset. Because there is no new structure, the number of parameter calculations of the improved model [24] is slightly lower than that of the original MobileNet model. A standard convolutional layer inputs the feature map *F* of $D_{F}$ × $D_{F}$ × *M*, and obtains an output feature map *G* of $D_{G}$ × $D_{G}$ × *N*, where DF represents the width and height of the input feature map, *M* is the number of input channels (input depth), *DG* is the width and height of the output feature map, *N* is the number of output channels (output depth), and *K* is the size of the depth convolution kernel. The table of parameters distributed in HDM-NET with classifier is (Table 1). The expression is as follows:

Depthwise convolutional parameters,

(5) $$D_{K}\times D_{K}\times M\times D_{F}\times D_{F}.\;$$

Pointwise convolutional parameters,

(6) $$M\times N\times D_{F}\times D_{F}.\;$$

Thus, all parameters are expressed as follows:

(7) $$P_{T}=D_{K}\times D_{K}\times M\times D_{F}\times D_{F}+M\times N\times D_{F}\times D_{F}.\;$$

## 3. Experiment Results

In the classification process, the most commonly used classifiers are SVM, multilayer perceptron (MLP), random forest (RF), K-nearest neighbors (KNN), radial basis function network (RBFN) [25], and Naive Bayes (NB) [26]. During the experiment, we conducted multiple sets of controlled trials. The four groups of experiments were compared and verified using the HDM-NET and SVM, HDM-NET and MLP, HDM-NET and RF, HDM-NET and KNN, MobileNet and SVM, MobileNet and MLP, MobileNet and RF, and MobileNet and KNN classification process. Compared to performance and accuracy of the HDM-NET+SVM architecture.

**Figure 6:**
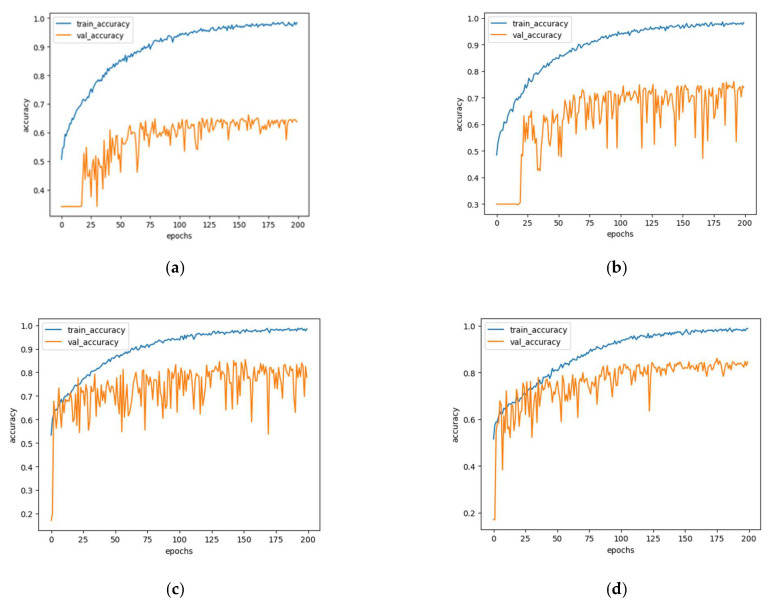
Accuracy of MobileNet (**a**) and HDM-NET (**b**) and the choice of attributes used for MobileNet (**c**) and HDM-NET (**d**).

For training, we used the Adam optimizer with a batch size of eight images, and a learning rate of 0.001 [27]. Because the image sizes collected in the original dataset are all 640 × 480, the optimizer will be slow during training and often lack video memory. Thus, the image is uniformly reduced to a pixel resolution of 224 × 224 and filled with zeros to maintain the original aspect ratio. A minimal data enhancement process was applied in the form of sample image rotation and rescaling. Apart from the standard pixel normalization of between zero and 1, no further preprocessing steps were applied to the input image. For experimental implementation, we used the Keras API in the Python3.6 TensorFlow2.1 framework. The training was conducted on an NVIDIA GeForce GTX 1660ti GPU equipped with 1536 CUDA cores, 6 GB of RAM, and a base clock speed of 1455 MHz. The comparative classification performance results shown in (Figure 6) show that, after testing on the same dataset DHI, the accuracy of HDM-NET is increased by 2.1% compared with the traditional CNN network. In particular, using our HDM-NET for feature extraction, combined with SVM, RF, MLP, and other methods for hair feature extraction and classification, the accuracy of the combined classification methods, such as Mobilenetv1, is generally higher. In many of the experiments, with HDM-NET combined with SVM, the final classification accuracy rate was the highest, reaching up to 94.8%.

Herein, we only used MobileNet and HDM-NET to complete the experiment. In addition, we did not use other CNN models for the experiments because such models (Table 2), such as VGG16 and GoogleNet, despite obtaining a high accuracy, have excessively large parameters for operation on mobile devices. Moreover, a small microscope was applied. The proposed model was improved and optimized on the basis of MobilenetV1, and, thus, we only conducted experiments for MobileNetV1 and HDM-NET. The proposed model has the least number of parameters, and its accuracy is slightly higher than that of the original MobileNet model. Because the MobileNet model has an ultra-small network framework, the classification accuracy is lower than that of the general CNN model. Our improved model based on MobileNet (HDM-NET) is far lower in accuracy than large-parameter models such as VGG16 (Table 3); however, compared with small networks such as MobileNet, our model has better classification results. Moreover, the parameters of the proposed model are small, and, thus, it is easier to directly complete the classification of hair damage images on a mobile microscope. We also used the HDM-NET and other models to complete the experiment (Table 4). We also tested and drew a conclusion of how long it took when one network was trained and when it had finished detecting certain images (Table 5). 

In this study, the structures of HDM-NET and SVM were used to analyze and classify the hair images collected under SEM. The HDM-NET architecture is proposed and a new SEM image dataset on hair was constructed separately to solve the limitations of other models in diagnosing hair images, including the following:

1. General CNN model training requires a large number of datasets, and the datasets we can use now are extremely small. 2. Using a larger model with too many parameters leads to poor training effects. 3. Although the pre-trained model obtained on ImageNet [33,34] can be used for migration learning to alleviate the results of a poor training effect owing to the lack of a dataset, in our survey, the ImageNet contains few SEM images of hair, and, thus, the effect is poor. Therefore, we can only choose a CNN model that has smaller parameters for improvement.

## 4. Conclusions

In this study, we proposed a fast and easy method for diagnosing the health of hair and proposed a lightweight deep learning framework. We completed the task of identifying and classifying our dataset extremely well. Although the final classification result is lower than that of the general CNN model, the accuracy is better than that of other lightweight CNN models when compared with models similar to our proposed lightweight CNN model (HDM-NET), e.g., SqueezeNet. In addition, the parameters are less than the original model. Owing to the compactness of the model, in the future, it can be easily transplanted to mobile phones or portable mobile devices [35] for conducting a hair diagnosis at any time. It will also promote other services such as hair care in the beauty industry. We have also proposed a new type of dataset on hair health, which we are still compiling. Deep learning provides convenience for fast hair health image diagnosis and classification tasks.

In general, this method is faster and more convenient than traditional physical and chemical diagnostic methods for hair health. At the same time, the high accuracy of the deep learning method shows the significant potential of deep learning in the diagnosis of hair health, and it can also be applied to micro or digital microscopes [36] (for example: SVBONY SM401 Microscope Wireless Digital Microscope 50×–1000×) connected to mobile phones in the future. Our experiments also proved that the attribute selection step in the feature selection process is beneficial for improving the accuracy of recognition and classification of microscope images.

## Figures and Tables

**Table 1:** Parameters distributed in HDM-NET with classifiers (SVM), dw means depthwise.

Layer Type	Stride	Kernel	Feature Map
Conv. Layer	2	3 × 3	3 × 32
Depthwise Conv.	1	3 × 3	32 dw
Conv. Layer	1	1 × 1	32 × 64
Depthwise Conv.	2	3 × 3	64 dw
Conv. Layer	1	1 × 1	64 × 128
Depthwise Conv.	1	3 × 3	128 dw
Conv. Layer	1	1 × 1	128 × 128
Depthwise Conv.	2	3 × 3	128 dw
Conv. Layer	1	1 × 1	128 × 256
Depthwise Conv.	1	3 × 3	256 dw
Conv. Layer	1	1 × 1	256 × 256
Depthwise Conv.	2	3 × 3	256 dw
Conv. Layer	1	1 × 1	256 × 512
2×Depthwise	1	3 × 3	512 dw
2×Conv. Layer	1	1 × 1	512 × 512
Depthwise Conv.	2	3 × 3	512 dw
Conv. Layer	1	1 × 1	512 × 1024
Depthwise Conv.	1	3 × 3	1024 dw
Conv. Layer	1	1 × 1	1024 × 1024
Global AvgPool	1	7×7	1024 × 1024
BN	---	---	1024 × 1024
ReLU	---	---	1024 × 1024
Classifier (SVM)	---	---	3

**Table 2:** Results of MobileNetV1 and HDM-NET on DHI datasets.

Model	Classifiers	Accuracy (%)
MobileNetV1	(Softmax)	85.3
MobileNetV1	SVM	92.6
MobileNetV1	RF	91.9
MobileNetV1	MLP	90.5
MobileNetV1	KNN	89.8
HDM-NET(ours)	(Softmax)	87.4
HDM-NET(ours)	SVM	94.8
HDM-NET(ours)	RF	91.4
HDM-NET(ours)	MLP	92.3
HDM-NET(ours)	KNN	90.3

**Table 3:** Parameters and results of general CNN models on DHI datasets.

Model	DHI Dataset Accuracy (%)	Million Parameters
VGG16	99.1	138 M
GoogleNet	97.2	6.8 M
ResNet50	98.6	25.5 M
SqueezeNetV1	83.4	1.2 M
ShufflenetV1	84.8	2.3 M
MobileNetV1	85.3	4.3 M
HDM-NET(ours)	87.4	1.4 M

**Table 4:** Results of HDM-net and other lightweight networks on DHI datasets.

Model	Classifiers	Accuracy (%)
SqueezeNetV1	(Softmax)	88.4
SqueezeNetV1	SVM	93.7
SqueezeNetV1	RF	87.3
SqueezeNetV1	MLP	90.2
SqueezeNetV1	KNN	89.8
ShufflenetV1	(Softmax)	86.5
ShufflenetV1	SVM	92.3
ShufflenetV1	RF	89.4
ShufflenetV1	MLP	90.6
ShufflenetV1	KNN	91.4
HDM-NET(ours)	SVM	94.8

**Table 5:** The training time and detection time for HDM-net and other networks (VGG16 [28], GoogleNet [29], ResNet50 [30]) and lightweight CNN models (SqueezeNetV1 [31], ShufflenetV1 [32], MobilenetV1).

Model	Training Time (hours)	Detection Time (s)
SqueezeNetV1	2.6	0.33
ShufflenetV1	2.6	0.52
MobileNetV1	2.7	0.55
VGG16	5.6	0.68
GoogleNet	3.1	0.46
ResNet50	3.4	0.51
HDM-NET(ours)	2.4	0.22

## Data Availability

The data are not publicly available due to privacy. The data presented in this study are available on request from the corresponding author.
